# Exploration of the Nutritional and Functional Properties of Underutilized Grains as an Alternative Source for the Research of Food-Derived Bioactive Peptides

**DOI:** 10.3390/nu15020351

**Published:** 2023-01-10

**Authors:** Samuel Fernández-Tomé, Tolulope Joshua Ashaolu, Blanca Hernández-Ledesma

**Affiliations:** 1 Department of Nutrition and Food Science, Faculty of Pharmacy, Complutense University of Madrid (UCM), Plaza Ramón y Cajal s/n, 28040 Madrid, Spain; 2 Institute for Global Health Innovations, Duy Tan University, Da Nang 550000, Vietnam; 3 Department of Bioactivity and Food Analysis, Institute of Food Science Research (CIAL, CSIC-UAM, CEI UAM+CSIC), Nicolás Cabrera 9, 28049 Madrid, Spain

**Keywords:** underutilized grains, functional foods, bioactive peptides, nutritional composition, enzymatic hydrolysis, gastrointestinal digestion, health benefits

## Abstract

The estimated increase in world population will lead to a deterioration in global food security, aggravated in developing countries by hidden hunger resulting from protein deficiency. To reduce or avoid this crisis, a dietary shift towards the consumption of sustainable, nutrient-rich, and calorically efficient food products has been recommended by the FAO and WHO. Plant proteins derived from grains and seeds provide nutritionally balanced diets, improve health status, reduce poverty, enhance food security, and contain several functional compounds. In this review, the current evidence on the nutritional and functional properties of underutilized grains is summarized, focusing on their incorporation into functional foods and the role of their proteins as novel source of bioactive peptides with health benefits.

## 1. Introduction to Underutilized Grains

In recent years, the continuous and concerning rise in world population and the consequent increase in food demand have challenged global food security. To aid with this global crisis, international organizations, governments, industries, and consumers are claiming sustainable, nutrient-rich and calorically efficient foods as an alternative or complement to animal products, and also reducing their associated negative-environmental impacts [1]. Food insecurity is higher in developing countries, where most people suffer from hidden hunger resulting from protein deficiencies [2]. This malnutrition problem can be addressed by exploring plant proteins as a sustainable and cost-affordable source of protein for a healthy diet [3]. Among plant proteins, those derived from grains and seeds have been recognized as the poor man’s meat, providing nutritionally balanced diets, improving health status, reducing poverty, and enhancing food security in low-income populations [4]. In addition to their nutritional role as a staple source of macro- and micro-nutrients, whole grain and seed proteins contain functional compounds such as bioactive peptides that play an important role in the prevention/management of various chronic disorders such as obesity, type 2 diabetes, cardiovascular disease, and cancer [5].

Grain- and seed-enriched proteins include wheat, brown rice, maize, millet, cornmeal, oatmeal, amaranth, buckwheat, couscous, teff, quinoa, flax, chia, and pumpkin, as well as sunflower seeds, peanuts, walnuts, almonds, cashews, dates, kiwi, and cumin. Cereal grains (*Gramineae* family) contain approximately 70–72% carbohydrates, 7–15% protein and 1–12% lipids, providing a significant source of energy in most diets when consumed as whole grains or in their refined form. Wheat, rice, and maize represent the major cereal crops sustaining over 50% of the worldwide caloric demand [6]. However, although these cereal grains constitute an important part of many diets, they are deficient in one or more essential amino acids as well as in some vitamins, minerals, and phytochemicals. To ensure access to nutritional grain-derived foods that meet human protein needs, approaches such as biofortification or supplementation have been implemented. On the other hand, in recent years, the interest has been focused on underutilized grains such as pseudocereals that traditionally have been used in rural areas as staple crops to provide essential amino acids, micronutrients and/or phytonutrients but the cultivation of which is limited and tied to cultural ancestry, and the agro-ecological and climate characteristics of their places of origin [7]. The present review examines the nutritional characteristics of underutilized grains as the basis for the development of functional foods, focusing on the role of their proteins as source of bioactive peptides with health benefits. The strategies used to release these peptides, as well as their demonstrated biological activities in both biochemical, and cell and animal models are also discussed.

## 2. Nutritional Value of Underutilized Grains as the Basis for the Development of Functional Foods

The nutritional composition of food provides values based on energy and is a measure of essential nutrients, including protein, carbohydrates, fat, fiber, vitamins, and minerals. Functional foods proffer healthful benefits beyond basic nutrient requirements due to their physiologically active components. It has been suggested that these foods support or contribute to the control of several health conditions [8]. Functional food products are being increasingly developed using several grains and seeds, which are usually consumed for their higher fiber and protein contents. Also, this is connected to the functional and nutritional properties of whole grains, which include the three critical parts of grains, that is, the bran, the germ, and the endosperm, as opposed to refined grains containing only the endosperm [9]. To go further within this perspective, Table 1 captures an updated overview of the nutritional properties, biological activity, and physicochemical and techno-functional features of underutilized grains incorporated into functional foods. The recent advances with respect to these attributes will be discussed below for each grain species, also considering the sensorial characteristics of the developed functional foods.

### 2.1. Amaranth

Amaranth is rich in various nutrients and bioactive molecules such as high-quality protein, unsaturated fats, dietary fiber, tocopherols, phenolic compounds, flavonoids, carotenoids, polyphenols, vitamins, and minerals. The elevated presence of phytochemicals makes amaranth a natural antioxidant-rich food [12]. Its leaves are rich in protein (17.9 g/kg), calcium (44.2 mg/100 g), iron (13.6 mg/100 g), zinc (3.8 mg/100 g), vitamin A (3.3 mg/100 g), and vitamin C (25.4 mg/100 g) on a dry weight basis [10]. Numerous health benefits have been linked to this pseudocereal, given its proposed hypo-cholesterolemic and anti-tumorigenic effects, immunomodulatory activity, anti-allergic action, impact on hepatic and glycemic function, and activity against celiac disease as a gluten-free product [15,46]. Its content in protein and various essential amino acids, especially lysine, are reportedly very high in comparison with other cereal grains [15].

This rich profile of amaranth has prompted a plethora of studies targeting its use in functional food products (Table 1). Cárdenas-Hernández et al. [17] reported a loss of antioxidant capacities in terms of ferric reducing antioxidant power (FRAP) and oxygen radical absorbance capacity (ORAC) after cooking amaranth-fortified elbow-type pasta, alongside the reduction of its luminosity values and the cooking time as compared to semolina pasta. The other merits of this development included the increase of protein, crude fiber, and ash contents in flour and dry leaves pasta, as well as iron, zinc, magnesium, and potassium in dry leaves pasta [17]. A more recent study showed that fortification of cassava pasta with amaranth could reduce cooking time and gruel solid loss while increasing its fiber, protein, β-carotene, iron, and zinc contents. Interestingly, the research was meant to present a healthy food choice to micronutrient-deficient consumers in low- and middle-income countries by combining a biofortified crop with leafy vegetables [10]. Earlier, semolina pasta enriched with digested and undigested amaranth proteins showed antihypertensive effects in a rat model, whereas it also reduced optimum cooking time and cooking loss [13]. However, the sensorial property was negatively impacted, especially the acceptability index. In the production of spaghetti pasta, a mixture of amaranth, sorghum, and wholesome sesame significantly increased protein, ash, fat, fiber, calcium, magnesium, and zinc, but also the contents of alkaloids, total phenolic, flavonoids, and 2,2′-diphenyl-1-picrylhydrazyl (DPPH) and FRAP activities relative to wheat flour, referred to as control pasta [21].

Apart from pasta kinds, cookies are popular types of food product intended for biofortification. In a study where enriched cookies were supplemented with hydrolyzed amaranth and fed to mice, angiotensin-converting enzyme (ACE) inhibition was found in serum, and the blood pressure of hypertensive rats was also reduced [11]. Hence, this study suggested the suitability of cookies as incorporation vehicles for amaranth antihypertensive hydrolyzates based on the enriched cookies’ ability to release in vivo ACE inhibitors that could pass through the murine bloodstream, while still presenting acceptable sensory properties. Following this research, a similar study in which functional cookies were enriched with amaranth showed an increase of total protein, ash, and flavonoid contents, including higher antioxidant capacity [12]. In addition, the microbiological quality and sensorial acceptability were not negatively affected. This premise is suggestive of amaranth flour being an optimum alternative for fortification of functional ingredients.

Puffed snacks and muffins are other functional foods that have been targeted with amaranth. Regarding their nutritional composition, their higher protein, iron, zinc, and dietary fiber, as well as increased total phenolics, flavonoid content and antioxidant (2,2′-azino-bis-(3-ethylbenzothiazoline-6-sulfonic acid) diammonium salt (ABTS), and DPPH) activities have been described in puffed snacks and muffins enriched with amaranth, respectively [14,15]. Moreover, the color and texture of the muffins were improved; this research developed out of the need for gluten-free functional foods to act against risks associated with celiac disease [15].

When it comes to bread, Olagunju and colleagues [16] reported that the nutritional status of multigrain bread improved after fortification with amaranth due to the increase in ash, fiber, and protein contents. Although the carbohydrate content was reduced, the product displayed hydroxyl radical scavenging ability, Fe^2+^ chelation, and inhibition of Fe^2+^-induced lipid peroxidation, coupled to an increase of α-amylase and α-glucosidase inhibitory abilities, and a decrease in glycaemic indices in comparison to white-flour bread [16]. Moreover, the combination of amaranth with other grains to produce functional bread could be significant. For example, when amaranth, quinoa, and chia were incorporated in fresh bread manufacturing, there was a significant increase in the protein, ash, and lipid contents [47]. The crumbs were also firmer than wheat bread, and the loaf-specific volume decreased, while similar calorie values were observed between the control and the fortified bread. Although the sensorial acceptability was similar between both products, the functional and nutritional values of the optimized bread were far superior to that of the control.

It is also possible to formulate beverages with amaranth proteins, as reported by two independent studies. In the study of Malgor and colleagues [18], amaranth lemon sorbet was developed directed to vegan and celiac consumers. These authors found, on one hand, increased foaming properties and, on the other hand, that antithrombotic peptides were released from amaranth protein after simulated gastrointestinal digestion of the beverage. Also, the functional sorbet rheology exerts the desired stability during a two-month storage, and it evinces acceptable sensory characteristics such as creaminess, airiness, and healthiness [18]. Amaranth-based beverages with good nutritional quality like that of skimmed milk with high-value proteins, soluble fiber, lipids, and carbohydrates have been also developed [19]. Amaranth proteins in the fractions of 11S and P globulins displayed the propensity of interacting to form aggregates and increase viscosity, thereby contributing to the physical stability of the beverage. Indeed, this functional drink exhibited more advantages than did plant-based milk products earlier developed [48].

### 2.2. Buckwheat

Belonging to the *Polygonaceae* family, buckwheat grains are rich in high flavonoids, dietary fiber, essential minerals, vitamins (e.g., B1, C, and E), and quality protein contents [49]. Apart from its gluten-free status, the proteins of buckwheat are rich in essential arginine and lysine amino acids and present excellent nutritional quality with high digestibility and bioavailability of amino acids. Also, buckwheat flavonoids such as rutin and quercetin exhibit high antioxidant activity [49]. This remarkable nutritional profile makes buckwheat protein a suitable source for functional food products.

Due to its protein, flavonoids, and rutin contents, buckwheat flour was used in bread production and found to increase iron content while the porosity and specificity of loaf volume became reduced [22]. Likewise, tartary buckwheat (*F. tartaricum*) and chia were combinedly used in bread manufacture resulting in an increased amount of protein, insoluble dietary fibers, ash, alpha-linolenic acid, and in vitro antioxidant power, parallel to reduced energy and carbohydrate contents [28].

Buckwheat has been also used to formulate functional biscuits. Zieliński and colleagues [23,24] performed extensive works regarding the utilization of buckwheat in functional biscuits aimed at inhibition of ACE and anti-advanced glycation end products (AGEs). They were able to characterize the total phenolic compounds that include *p*-coumaric, sinapic, syringic, vanillic, protocatechuic acids, kaempherol, quercetin, apigenin, and orientin, and could partially correlate ACE inhibitory activity with the content of total phenolic compounds. Indeed, they observed a decrease in ACE inhibition, which was reverted by simulated gastrointestinal digestion, whereas higher anti-AGEs activity, antioxidant/reducing capacity and total phenolics contents were found following simulated digestion of biscuits [23,24]. It is thus inferred that the bio-accessible phenolic antioxidant compounds contributed to the inhibitory activity of biscuits against AGEs formation. An increase in dietary fiber content and antioxidant capacity has been also reported in functional deserts in which buckwheat by-products and derived melanin were incorporated [27]. Further complementing the functional role of this grain, it has recently been shown that tartary buckwheat supplementation for 12 weeks can attenuate serum oxidative and inflammatory responses, modulate gut microbiota and colonic short-chain fatty acids, and improve lipid metabolism of high-fat-diet-fed rats, like the effect displayed by an oat-supplemented diet [50].

Common buckwheat (*F. esculentum* Moench) and tartary buckwheat were comparatively studied for their cooking, textural, sensorial, and antioxidant activities in the noodling process [26]. They showed low and high concentrations of total phenolics and flavonoids in both grain sources, respectively, and correspondingly exhibition of low and high antioxidant capacities (FRAP, ABTS, and DPPH). On the other hand, the common buckwheat made the noodles’ tensility higher, but reduced adhesiveness whereas tartary buckwheat offered lower tensility and increased adhesiveness [26].

Functional beef burgers incorporated with buckwheat and quinoa showed an increase in magnesium, phosphorus, iron, and zinc contents in comparison with control burgers while their oil absorption and water holding capacity were significantly reduced when compared with soy protein and bread crumb-based control burgers [25]. Although the control burgers had higher protein and ash contents and lower carbohydrate content, the quinoa and buckwheat burgers showed higher levels of several minerals, increased sensory acceptance, and increased shelf-life stability [25]. Authors suggested that the replacement of soy protein and bread crumbs with buckwheat and quinoa flour is feasible when formulating beef burgers with improved nutritious and sensorial qualities, while gluten-sensitive and celiac consumers may obtain a beef burger alternative.

### 2.3. Chia

Chia (*S. hipanica* L.) grains are included in the group of alternative food products that have attracted the interest of both consumers and the food industry in recent years because of its nutritional quality, functional effects, and proposed health benefits ranging from cardiovascular health to chemopreventive properties against chronic disorders. Chia belongs to the *Lamiaceae* family, and its seeds have high dietary fiber (35%), lipids (40%), high-quality protein (19%), natural minerals, antioxidants, vitamins, and polyphenol and tocopherol contents [51].

Due to its rich nutritional profile, Alcântara Brandão et al. [29] reported that chia, as well as its seed blends and derived flours, increased the protein and dietary fiber contents of manufactured functional cookies, sensorial properties and acceptability of which were positively affected at purchase intention. A year later, sweet cookies incorporated with defatted chia flour were observed to increase their polyphenol content and antioxidant capacity (FRAP and ABTS) [34]. In this study, although lesser amounts of polyphenols were released from the food matrix during gastrointestinal digestion and absorbed through the intestinal barrier by passive diffusion, there was a greater release of polyphenols and antioxidant activity during colonic fermentation, thus establishing its potential prebiotic effects [34].

The richness of polyphenols and polyunsaturated fatty acids (PUFA) in chia cannot be diminished. In the formulation of beef burgers, chia seeds and goji puree were evaluated for their effects on the fatty acid profile, lipid peroxidation, total phenols, and antioxidant capacity of cooked beef burgers [30]. An increase in polyphenol bio-accessibility and antioxidant capacities (ORAC, ABTS, DPPH), as well as a reduction of malondialdehyde (MDA) values were found after simulated gastrointestinal digestion.

The use of chia in bread manufacture is another area for functional food development. Recently, chia seeds and kinako flour were utilized in the improvement of lipid profile in bread [31]. The authors reported an increase in PUFA content, with special attention to linolenic acid, and reduced contents of saturated fatty acid (SFA) and monounsaturated fatty acids (MUFAs). Similar results were obtained for incorporation of chia flour, which provided an increased amount of linoleic acid, protein, lipids, and minerals content and a reduced glycaemic index in comparison to wheat-based bread [20]. Moreover, Verdú et al. [37] studied the techno-functional effects of chia seed flour on the bread-making process and found a reduction of water activity and an equal amount of moisture compared to the control along with an improvement of gas retention in the dough, thereby cutting the time required to reach maximum dough development.

Functional yogurts are popular among consumers of functional foods. Hence, the study of Kowaleski et al. [32] developed yogurt formulations with enhanced nutritional properties and favoring probiotic effects by using strawberries and chia seeds supplementation. Earlier, another group of researchers used chia seed extract to fortify yogurt, and its radical scavenging activity and protection against lipopolysaccharide (LPS)-induced damage was increased in human colon cells [33]. Moreover, these in vitro effects were demonstrated in parallel to the acceleration of fermentation rate and growth of lactic acid and improvement of syneresis and water-holding capacity [33]. Hence, it is suggested that a set type of yogurt can benefit from chia supplementation, which enhances the growth of lactic acid bacteria, the physicochemical properties, and the health benefits of the final product.

Other functional food ingredients and products such as pineapple jam [35], cake [36], kulfi dessert [38], corn tortillas [39], and gluten-free noodles [40] have been also formulated or developed whilst incorporating chia or its by-products. From these studies, it may be inferred that adding chia components to a set type of functional products allows the enhancement of their nutritional profile, physicochemical properties, and the health benefits of the final product (Table 1). As an example, gluten-free noodles were developed with increased protein, fat, phytic acid, and phytate phosphorus contents along with several micronutrients, while there was also an increase in antioxidant activity, total phenolic content, and a significant rise in volume and weight.

### 2.4. Lupin

Lupins (*Lupinus albus* L.) are distinguished by their high-quality protein and dietary fiber contents, which are associated with cholesterol-lowering activity. Their seeds are enriched with high levels of total unsaturated fatty acids and contain vitamins such as thiamine, niacin, riboflavin, and tocopherols, as well as minerals including iron, zinc, and manganese [41]. When processed into flour and protein isolates, lupins are rich in antioxidants like polyphenolic tannins and flavonoids [52]. When compared with soybean and other legumes, lupins have lower levels of anti-nutritional components such as phytate and saponins. Besides, the amounts of lysine, isoleucine, leucine, phenylalanine, and tyrosine in lupin are in the optimal range of reference protein for adults according to the Food and Agricultural Organization (FAO) standards [41].

Studies summarized in Table 1 have found that proteins and dietary fiber contents of lupins have the potential to increase the nutritional quality and modify the technological properties of bread and other baked products when wheat flour is supplemented by lupin flour. This serves as the backdrop for the supplementation of wheat flour with lupin flour in biscuits production [41]. The authors reported an increase in protein, lipid, fiber, and ash contents, and a reduction in carbohydrate content compared with the control biscuits made of wheat flour. There was also a rise in all amino acid content, and increased quality scores.

Lupin flour can be used to formulate both functional baked products [53] and other non-baked foods like dairy products [54], pasta [55], and meat products [56]. Hence, in beef sausage lupin incorporation, increased dietary fiber and reduced fat content were reported after cooking [43]. Moreover, the meat emulsion stability increased, the cooking loss decreased, and there was a greater adhesiveness in the final product. In sweet-cookie formulation with lupin seed extract, low values of water activity and moisture content were obtained while higher firmness but a reduced impact on the shape parameters as well as lightness were observed in the finished cookies [42].

### 2.5. Quinoa

Quinoa (*Chenopodium quinoa*) has been grown for more than five millennia and it is a good source of starch, vitamins, minerals, protein, fat, dietary fiber, and polyphenols [57]. Quinoa seed has a high protein quality (average percentage~14 to 18%) due to its elevated concentrations of lysine and histidine amino acids, while is a rich source of digestible plant protein for celiac and gluten-sensitive consumers [58]. It also contains high amounts of micronutrients including magnesium, calcium, zinc, copper, iron, niacin, and α- carotene [25].

In functional bread development, quinoa flour has been used with the result of increased yellowness and increased contents of vitamins, minerals, essential amino acids, protein, dietary fiber, Mg, Fe, and P, while reducing the starch content, lightness, and redness of the finished products [44,45]. The implication is that both nutritional qualities and the sensory and physico-chemical properties of bread may be enhanced by the incorporation of quinoa and its products. Following this line of thought, a mix of quinoa and buckwheat flour was used for the formulation of functional beef burgers [25]. This study found increased shelf-life stability, increased contents of magnesium, phosphorus, iron, and zinc, as well as reduced oil absorption and water holding capacity, in comparison with control burgers made of soy protein and breadcrumbs.

## 3. Sensorial Properties of Underutilized Grains-Based Functional Foods

**Figure 1:**
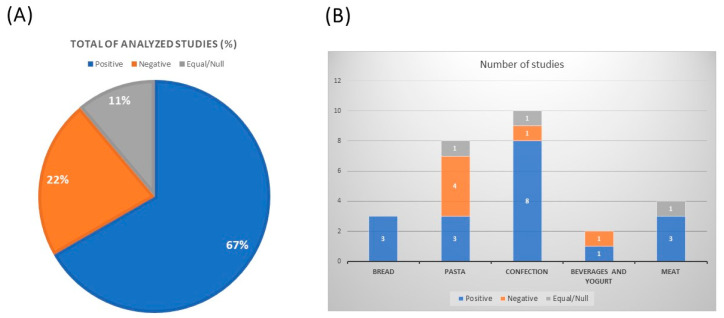
Sensorial analysis of functional food products developed by incorporation of underutilized grains. (**A**) Percentage of the analyzed studies. A total of 27 studies that evaluated the sensorial analysis of developed products were summarized in this section of the review from the literature search (PubMed and Web of Science). The reported sensory attributes were categorized as positive, negative, or null outputs in the final formulation. (**B**) Classification of the resulting sensorial attributes in each food group including bread, pasta, confectionary, meat, beverages, and yogurt.

Underutilized grains have been thus incorporated into a variety of food products aimed at improvement of their final nutritional, techno-functional and bioactive properties. Nonetheless, consideration of the sensorial attributes of the final product is noteworthy; they are a key aspect for consumers’ acceptance of functional foods. Indeed, some of the previous studies performed complementary sensorial analysis of developed products, thereby contemplating whether the incorporation of these grains resulted in a positive/negative/null impact on the formulation (Figure 1A). Table 2 further details the sensorial outputs of various functional food types including staple foods such as bread, pasta, dairy, meat, and confectionery, as well as beverages.

From the literature summarized in the present study, data suggest that positive sensory attributes were obtained in most of the cases (67%, 18 out of the 27 studies). As some examples, optimal sensorial characteristics included good scores for bread quality indicators [22], improvement of taste and odor in noodles [40], color intensification of enriched cookies [11], and increased overall acceptability and taste of supplemented meat products [25]. On the contrary, up to one-third of the studies described a null impact or negative outcomes, such as bitter taste [26], reduced luminosity values [17], or even a deleterious effect on the overall acceptability of the food product [13,35].

Interestingly, the present review has found that confectionery items compiled the group of products with more positive entries in the sensorial analysis, while most of the negative outcomes have been reported in the case of grain-supplemented pasta products (Figure 1B). It has been also found that the grain species, and the level of supplementation and/or the co-supplementation with other food compounds may impact the final product characteristics. Optimal sensory attributes of noodles were found for common buckwheat with good cooking qualities, but not in the case of noodles based on tartary buckwheat, which showed much poorer sensorial qualities [26]. The study of Kowaleski and colleagues in yogurts indicated that sensory acceptance was inversely proportional to the addition of chia but increased with strawberry addition. Hence, yogurt containing 6% chia and 12% strawberry had an acceptability index >70%, considered as the limit for a product to be sensorially acceptable, and therefore identified as the best formulation of their functional study due to its higher nutritional value [32]. Baked products such as gluten-free cakes and muffins found that lupin flour incorporation is possible, at levels of up to 20–30%, without negatively affecting the product’s sensorial qualities and consumer acceptance [59].

## 4. Production of Bioactive Peptides Derived from Underutilized Grains and Study of Their Health Effects

One of the benefits reported for underutilized grains are those associated with their protein component as novel source for bioactive peptides. On this basis, many reports have supported the idea that hydrolyzates and bioactive peptides produced from underutilized grains may provide healthful outcomes. The production technique, the degree of hydrolysis, the chain length of peptides and their physicochemical properties, as well as the amino acid profile will affect the activity of the resultant hydrolyzates. To release bioactive peptides from underutilized grains, the enzymatic hydrolytic method is an interesting alternative to solvent extraction, fermentation, and chemical treatments, as no residual organic solvents or toxic chemicals are released in the final products [60].

Once the grain protein is treated with various proteases, broad hydrolyzates and peptides are produced, and may then be purified to attain and characterize specific bioactive peptides. Hence, the sequence of amino acids and chain lengths become altered after hydrolysis, creating novel functionalities, an attestation to the fact that different proteases produce different types of peptides from the same protein substrate [61,62]. Therefore, the hydrolytic reaction can be well-regulated to accurately ascertain the peptides’ release, their molecular sizes, sequences, amino acid profile, hydrophobicity, and their potential mechanism of action and physiological effects. Gastrointestinal digestion is another common strategy used at obtaining hydrolyzates and peptides from grains. Following this strategy, the advantages are therefore like those of enzymatic treatments, as no toxic residues are involved. Table 3 summarizes a plethora of studies using these two most common methods of protein hydrolysis to ascertain the bioactivities of hydrolyzates and/or peptides from selected underutilized grains.

### 4.1. Enzymatic Protein Hydrolysis

#### 4.1.1. Amaranth and Quinoa Hydrolyzates

The protein component of amaranth makes this grain an ideal candidate for peptides exploitation. Alcalase, bromelain, chymotrypsin, trypsin, or protease have been repeatedly used in this process with observable activities reported after in vitro, in vivo, and ex vivo studies. For example, antimicrobial, ACE- and dipeptidyl peptidase (DPP)-IV-inhibitory, antihypertensive, antihaemolytic, anti-inflammatory, renin-inhibitory, radical-scavenging, and apoptotic activities of amaranth protein hydrolyzates have been reported [63,64,65,66,67,68,69,70,71,72].

Specific sequences such as the fragment SSEDIKE have been associated with anti-inflammatory activity in human intestinal epithelial cells [73]. Likewise, QAFEDGFEWVSFK, AFEDGFEWVSFK, SFNLPILR, FNLPILR, SFNLPIL, and VNVDDPSKA amaranth peptides showed renin-inhibitory activity in biochemical assays due to their hydrophobic interactions [69], while a group of di-, tri-, and tetra-peptides from amaranth glutelin induced endothelial nitric oxide (NO) production in coronary endothelial cells along with ACE inhibitory action [65].

Alcalase-treated quinoa peptides with a molecular weight lower than 5000 Da could confer ACE inhibition and radical scavenging activities [74]. However, a series of studies addressing the bioactivities of quinoa hydrolyzates by biochemical assays did not showcase the specific peptides responsible for the reported effects (Table 3). Nonetheless, they did demonstrate that alcalase, pancreatin, pepsin, corolase, bromelain, chymotrypsin, or protease-treated proteins from quinoa may display radical (ABTS and DPPH) scavenging activities [71,74,75,76,77]. The hydrolyzates obtained through the treatment with bromelain, chymotrypsin, or protease exerted not only radical scavenging, but also antimicrobial (against *Staphylococcus aureus*, *Salmonella typhimurium*, *Escherichia coli*, and *Enterobacter aerogenes*), and antihaemolytic activities in human erythrocytes [71].

**Table 3:** Evaluation of production and health effects of proteins, hydrolyzates and bioactive peptides derived from underutilized grains.

Production of Peptides	Grain Specie	Proteins/Peptides		Bioactivity		Reference
		Sample	Responsible Sequence	Biochemical Assay	Cell-Based and Animal Models	
Enzymatic protein hydrolysis	**Amaranth**	Alcalase hydrolyzate	---	---	Antihypertensive activity in SHR	[72]
		Hydrolyzates by bromelain, chymotrypsin, or protease	---	Radical (ABTS, DPPH) scavenging activity Antimicrobial activity against *S. aureus*, *S. typhimurium*, *E. coli*, and *E. aerogenes*	Non-haemolytic activity in human erythrocytes	[71]
		Alcalase hydrolyzate	---	ACE inhibitory activity	Antihypertensive activity in SHR	[70]
		Trypsin hydrolyzate of glutelin fraction	Di-, tri-, and tetra-peptides	ACE inhibitory activity	Induction of endothelial NO production in coronary endothelial cells	[65]
	Amaranth (*A. hypochondriacus*)	Alcalase hydrolyzate of albumin I and globulin fractions	---	ACE inhibitory activity	---	[64]
		Alcalase hydrolyzate of albumin and globulin fractions	---	DPP-IV inhibitory activity	Control of postprandial glycemia in STZ-induced diabetic mice	[67]
		Alcalase hydrolyzate	SSEDIKE	---	Anti-inflammatory (reduced activation of human intestinal epithelial cell) activity	[68]
		Trypsin hydrolyzate of glutelin fraction	Multiple sequences	Multifunctionality	Induction of apoptosis in HeLa cells	[63]
		Alcalase hydrolyzate	QAFEDGFEWVSFK, AFEDGFEWVSFK, SFNLPILR, FNLPILR, SFNLPIL, and VNVDDPSKA	Renin inhibitory activity related to peptide hydrophobicity	---	[69]
	Amaranth (*A. mantegazzianus*)	Alcalase hydrolyzate	---	ACE inhibitory activity	Antihypertensive activity in SHR	[66]
	Quinoa (*C. quinoa* Willd.)	Alcalase hydrolyzate	Peptides with MW lower than 5000 Da	ACE inhibitory activity Radical (DPPH) scavenging activity	---	[74]
		Hydrolyzates by bromelain, chymotrypsin, or protease	---	Radical (ABTS, DPPH) scavenging activity Antimicrobial activity against *S. aureus*, *S. typhimurium*, *E. coli*, and *E. aerogenes*	Non-haemolytic activity in human erythrocytes	[71]
		Pancreatin hydrolyzate	---	Radical (DPPH) scavenging activity	---	[77]
		Pepsin and alcalase hydrolyzates	---	Radical (ABTS) scavenging activity	---	[75]
		Corolase^®^ 7089 hydrolyzate	---	Radical (ABTS) scavenging activity	---	[76]
	Chia (*S. hispanica*)	Alcalase and Flavourzyme hydrolyzates	---	ACE inhibitory activity Radical (ABTS) scavenging activity Antimicrobial activity against *E. coli*, *S. typhi*, *S. flexneri, K. pneumonia*, *S. aureus*, *B. subtilis* and *S. agalactae*	---	[78]
		Alcalase and alcalase + flavourzyme hydrolyzates	Fraction lower than 3 kDa	Antimicrobial activity towards *E. coli*, *S. enterica*, and *L. monocytogenes*	---	[79]
		Alcalase + flavourzyme hydrolyzates	---	Antioxidant (β-carotene discoloration) activity FRAP Iron and copper chelating capacity	---	[80]
		Papain hydrolyzate	---	Radical (ABTS, DPPH) scavenging activity	---	[81]
		Pepsin + pancreatin hydrolyzate	Fraction lower than 3 kDa	---	Neuroprotective effect on H_2_O_2_-induced damage on N1E-115 cells	[82]
		Alcalase, pepsin, trypsin, or α-chymotrypsin hydrolyzates	LIVSPLAGRL	ACE inhibitory activity	---	[83]
		Alcalase, flavourzyme and sequential alcalase-flavourzyme hydrolyzates	Fraction lower than 3 kDa	Antimicrobial activity against *S. aureus* Hypocholesterolemic activity through inhibition of HMG-CoA reductase	---	[84]
		Alcalase-flavourzyme and pepsin-pancreatin hydrolyzates	Fractions 5–10 kDa and higher than 10 kDa	α-amylase and α-glucosidase inhibitory activity	---	[85]
		Alcalase and sequential alcalase-flavourzyme hydrolyzates	---	ACE inhibitory activity Radical (ABTS, ORAC) scavenging activity Metal chelating capacity DPP-IV inhibitory activity	Inhibition of ROS generation in oxidized-induced Caco2 cells	[86]
		Alcalase or flavourzyme hydrolyzates	---	ACE inhibitory activity Antioxidant (β-Carotene-linoleic acid assay) activity Radical (DPPH) scavenging activity FRAP	---	[87]
	Tartary buckwheat (*F. tataricum* Gaertn.)	Albumin hydrolyzate by alkaline protease	GEVPW, YMENF, and AFYRW	Radical (OH, DPPH) scavenging activity Lipid peroxidation inhibitory activity FRAP	---	[88]
		Enzymatic hydrolyzates of 13S globulin acidic subunit	SEAGVTEIWDHNTPEFR, CTGFVAVR, YVIQPGGLLLPSYSNAPYITFVEQGR, SFFLAGQSQQGR, LRGENDQR, and GFIVQAQDLK	---	Maintenance of the redox state balance of HepG2 cells Protection of the activity of antioxidant cell enzymes via the PPAR-α/HO-1 pathway	[89]
		Alcalase hydrolyzate	---	α-amylase, and α-glucosidase inhibitory activity	---	[90]
	Chickpea (*C. arietinum* L.)	Papain or ficin hydrolyzates from germinated chickpea protein	SPGAGKG, GLAR, and STSA	DPP-IV, and α-glucosidase inhibitory activity	---	[91]
		Alcalase hydrolyzates from albumin and globulin fractions	FEI, FEL, FIE, FKN, FGKG, and MEE	Radical (ABTS, DPPH) scavenging activity DPP-IV inhibitory activity	---	[92]
		Alcalase hydrolyzates	VFVRN	---	Hypolipidemic activity in high fat diet-induced obese rats	[93]
	Lupin	Alcalase or flavourzyme hydrolyzates	---	Radical (ABTS, DPPH) scavenging activity	---	[94]
		Alcalase, trypsin or pepsin hydrolyzates	---	ACE inhibitory activity Radical (ABTS, DPPH) scavenging activity FRAP	Protection against oxidative stress in HepG2 cells	[95]
	Lupin (*L. angustifolius* L.)	Alcalase hydrolyzate	---	---	Antioxidant and anti-inflammatory effects in LPS-stimulated THP-1 cells and co-cultures of Caco-2 and THP-1 cells. Reduction of ROS, nitric oxide, and pro-inflammatory cytokines levels	[96]
		Hydrolyzate	GPETAFLR	---	Protective role on oxidative and inflammatory markers involved in age-related macular degeneration in RPE cells	[97]
	Pearl millet (*P. glaucum*)	Trypsin hydrolyzate	SDRDLLGPNNQYLPK	Radical (ABTS, DPPH, OH) scavenging activity Iron chelating ability FRAP	---	[98]
	Finger millet	Pepsin or trypsin hydrolyzates	TSSSLNMAVRGGLTR and STTVGLGISMRSASVR	Radical (ABTS, DPPH, OH) scavenging activity Iron chelating ability	---	[99]
	Mung bean (*V. radiata* L. Wilczek)	Hydrolyzates by alcalase, flavourzyme, trypsin, pancreatin, pepsin, pancreatin + alcalase, and pancreatin + flavourzyme	LLLGI, AIVIL, HADAD, and PAIDL	Calcium and iron-binding activity	---	[100]
		Bromelain hydrolyzates	LPRL, YADLVE, LRLESF, HLNVVHEN, and PGSGCAGTDL HC, CGN, LAN, CTN, LAF, CSGD, MMGW, QFAAD, ERF, EYW, FLQL, and QFAW	ACE and renin inhibitory activity Radical (DPPH) scavenging activity Iron chelating ability FRAP	Antihypertensive activity in SHR	[101,102]
		Alcalase, neutral protease, or papain hydrolyzates	---	Iron chelating ability	---	[103]
	Green tender sorghum	Alcalase hydrolyzate	VPPSKLSP, VAITLTMK, GLLGKNFTSK, LDSCKDYVME, HQTSEFK, VSKSVLVK, and TSVEIITSSK	Radical (DPPH, ABTS) scavenging activity Iron chelating ability FRAP	---	[104]
	White sorghum (*S. bicolor* L.)	Alcalase hydrolyzate from kafirin fraction	Fractions lower than 3 and 1 kDa	---	Antioxidant, anti-inflammatory, and anti-aging protection in ultraviolet B irradiated-human organotypic skin cultures	[105]
	Sweet sorghum (*S. bicolor* L.)	Alcalase hydrolyzate	TLS	ACE inhibitory activity	---	[106]
	White sorghum (*S. bicolor* L.)	Papain hydrolyzate from kafirin fraction	LRQQ, QLQGV, WQPN, GLQDL, LRQQ, QLQGV, WQPN, AMCGVV, YLRQ, TPCATS, QGVAAA, AQVAQ, and QQLQ	Radical (DPPH, ABTS, ORAC) scavenging activity Iron chelating ability FRAP	Chemopreventive effects in HepG2 cells	[107]
Gastrointestinal digestion of proteins	Amaranth (*A. caudatus*)	Simulated gastrointestinal digests	YESGSQ, GGEDE, NRPET, FLISCLL, TALEPT, HVIKPPS, SVFDEELS, DFIILE, and ASANEPDEN	ACE inhibitory activity Radical (ORAC) scavenging activity DPP-IV and α-amylase inhibitory activity	Colon cancer cell viability inhibitory activity	[108]
		Simulated gastrointestinal digests	---	Radical (DPPH) scavenging activity	Anti-cancer activity against breast cancer MDA-MB-231 cells	[109]
		Simulated gastrointestinal digests of Alcalase hydrolyzate	---	ACE inhibitory activity	---	[110]
	Amaranth (*A. hypochondriacus*)	Simulated gastrointestinal digests of trypsin hydrolyzate	STHASGFFFFHPT, GLTEVWDSNEQEF, STNYFLISCLLFVLFNGCMGEG, and TIEPHGLLLPSFTSAPELIYIEQGNGITGMMIPGCPETYESGSQQFQGGEDE	DPP-IV inhibitory activity	---	[111]
		Simulated gastrointestinal digests	---	ACE inhibitory activity DPP-IV inhibitory activity	---	[112]
		Simulated gastrointestinal digests of sprouts protein	---	ACE inhibitory activity Radical (ABTS, ORAC) scavenging activity	---	[113]
		Simulated gastrointestinal digests of germinated amaranth	Multiple sequences	Radical (ORAC) scavenging activity	Anti-inflammatory activity in RAW264.7 cells	[114]
	Amaranth (*A. mantegazzianus*)	Simulated gastrointestinal digests	---	---	Antiproliferative activity in human colon cancer cells	[115]
	Quinoa (*C. quinoa* Willd.)	Simulated gastrointestinal digests	IQAEGGLT, DKDYPK, and GEHGSDGNV	DPP-IV, α-amylase, and α-glucosidase inhibitory activity	---	[116]
		Simulated gastrointestinal digests	FHPFPR, NWFPLPR, and NIFRPF	ACE inhibitory activity	Antihypertensive activity in SHR	[117]
	Chia (*S. hispanica*)	Simulated gastrointestinal digests of albumin and globulin fractions	---	ACE inhibitory activity Radical (ABTS, DPPH) scavenging activity Ferrous chelating activity	---	[118]
	Buckwheat (*F. esculentum*)	Simulated gastrointestinal digests and trypsin and alcalase hydrolysis of albumin and glutelin fractions	---	DPP-IV inhibitory activity	---	[119]
		Simulated gastrointestinal digests and trypsin and alcalase hydrolyzates	---	Inhibition of platelet aggregation	---	[120]
	Chickpea (*C. arietinum* L.)	Simulated gastrointestinal digests and bromelain hydrolyzates	PHPATSGGGL and YVDGSGTPLT	DPP-IV, α-amylase, and α-glucosidase inhibitory activity	---	[121]
	Cocoa (*T. cacao* L.)	Simulated gastrointestinal digests of cocoa seeds proteins	LSPGGAAV, TSVSGAGGPGAGR, and TLGNPAAAGPF	ACE inhibitory activity	Antihypertensive activity in a high-fat diet rat model	[122]
	Foxtail millet (*S. italica*)	Simulated gastrointestinal digests of germinated and heated foxtail millet proteins	SEDDPFD, REEGVLIF, EAGNKGTLSF, MGPIPSTL, EDDQMDPMAK, QNWDFCEAWEPCF, and MSHRGACGCEK	---	Antioxidant and anti-inflammatory effects in Caco-2 cells	[123]
	Mung bean (*V. radiata* L. Wilczek)	Simulated gastrointestinal digests and thermolysin hydrolyzates	Multiple potential sequences of di- and tri-peptides	Radical (ABTS, ORAC) scavenging activity Iron chelating ability	---	[124]
Other strategies	Amaranth	Fermentation with *L. casei* Shirota and *S. thermophilus* 54,102 in mono and combined culture	---	ACE inhibitory activity Radical (ABTS, DPPH) scavenging activity and FRAP Antithrombotic (thrombin inhibition) activity	---	[125]
	Amaranth (*A. mantegazzianus*)	Protein isolate	---	---	Reduction of plasma and liver cholesterol levels in rats Increment in FRAP values, diminution of TBA value in plasma and liver, and SOD activity in plasma Decrease of blood pressure	[126]
	Tartary buckwheat (*F. tataricum* Gaertn.)	Peptide obtained by gene cloning and expression	GSSEKPQQELEECQNVCRMKRWSTEM-VHRCEKKCEEKFERQQR	Inhibition of trypsin activity Antifungal activity against *T. koningii*, *Rhizopus* sp., and *F. oxysporum*	---	[127]
	Buckwheat (*F. esculentum*)	Purification of 11 kDa protein from buckwheat seed extract	N-terminal sequence AQXGAQGGGAT	Antifungal activity against *B. cinerea*	---	[128]

ABTS: 2,2′-azinobis(3-ethylbenzothiazoline-6-sulphonic acid) diammonium salt; ACE: angiotensin converting enzyme; DPPH: 2,2-Diphenyl-1-picrylhydrazyl; DPP-IV: dipeptidyl peptidase-IV; FRAP: ferric reducing antioxidant power; HDL-C: high-density lipoprotein cholesterol; HMG-CoA reductase: 3-hydroxy-3-methylglutaryl coenzyme A reductase; LDL-C: low-density lipoprotein cholesterol; ORAC: oxygen radical antioxidant capacity; SHR: spontaneously hypertensive rats; STZ: streptozotocin.

#### 4.1.2. Chia Hydrolyzates

The chia protein hydrolyzates offer a wide range of biological benefits ranging from radical scavenging (ABTS, DPPH, and ORAC), metal chelating, and antimicrobial actions to ACE inhibition, hypocholesterolemic, antidiabetic, and antioxidant activities, among others (Table 3).

San Pablo-Osorio et al. [83] reported that the sequence LIVSPLAGRL identified in different enzymatic chia hydrolyzates inhibited the activity of ACE. While this peptide was isolated as the responsible sequence, other reports so far have only explored chia hydrolyzates fractions for biological studies. An instance is the obtaining of 5–10 kDa and ˃10 kDa fractions after treatment of chia protein with alcalase-flavourzyme and pepsin-pancreatin enzyme complexes [85], which showed antidiabetic potential as demonstrated by *α*-amylase and *α*-glucosidase inhibitory activities.

Recently, three different groups of researchers used fractions lower than 3 kDa obtained by the application of different enzymes for their bioactivity studies. The first group used alcalase, flavourzyme, and sequential alcalase-flavourzyme in the production of chia hydrolyzates with the capacity of displaying antimicrobial activity against *S. aureus* and hypocholesterolemic effect through inhibition of β-hydroxy β-methylglutaryl-CoA (HMG-CoA) reductase [84]. The other group reported that chia hydrolyzates produced with alcalase and alcalase-flavourzyme showed antimicrobial activity against *E. coli*, *Salmonella enterica*, and *Listeria monocytogenes* [79]. Lastly, Martínez Leo & Segura Campos [82] demonstrated that the treatment of chia protein with pepsin and pancreatin was able to generate peptides with neuroprotective effects against H2O2-damage in N1E-115 cells.

#### 4.1.3. Buckwheat and Chickpea Hydrolyzates

The buckwheat protein, specifically albumin, hydrolyzed by alkaline protease, and the isolated peptides GEVPW, YMENF, and AFYRW, possessed radical scavenging, metal chelating, and lipid peroxidation inhibitory effects [88]. Earlier, alcalase hydrolyzates of buckwheat were also found to display *α*-amylase and *α*-glucosidase inhibitory activity [90]. However, in another recent study, some buckwheat peptides were obtained following the enzymatic hydrolysis of 13S globulin acidic subunit, namely SEAGVTEIWDHNTPEFR, CTGFVAVR, YVIQPGGLLLPSYSNAPYITFVEQGR, SFFLAGQSQQGR, LRGENDQR, and GFIVQAQDLK. These fragments exerted antioxidant protection in the hepatic cell line HepG2 by maintaining the redox state balance and the activity of antioxidant cell enzymes via the PPAR-α/HO-1 pathway [89].

Very recently, chickpea peptides, namely SPGAGKG, GLAR, and STSA were identified after hydrolysis of germinated chickpea protein with papain or ficin [91]. These peptides showed DPP-IV and *α*-glucosidase inhibition. The antidiabetic and radical scavenging potential was also reported for peptides FEI, FEL, FIE, FKN, FGKG, and MEE derived from the albumin and globulin fractions of alcalase-treated chickpea protein [92]. In vivo, the alcalase hydrolyzates of chickpea protein and peptide VFVRN demonstrated hypolipidemic activity in high-fat-diet-induced obese rats [93].

#### 4.1.4. Lupin and Millet Hydrolyzates

The synthetic peptide GPETAFLR, identified from lupin protein hydrolyzate, could play a preventive role against age-related macular degeneration as demonstrated by in vitro experiments in RPE cells and determination of protection of oxidative and inflammatory markers [97]. However, some other studies on lupin hydrolyzates have not reported the specific peptides involved in the biological functions. For example, alcalase or flavourzyme hydrolyzates of lupin showed radical scavenging activity, as well as antioxidant and anti-inflammatory effects in LPS-stimulated THP-1 cells and Caco-2 and THP-1 co-cultures [94,96]. Alcalase, trypsin, and pepsin hydrolyzates exerted ACE inhibitory, radical (ABTS, DPPH) scavenging, and metal (FRAP) chelating activities in non-cellular systems, apart from protecting hepatic cells against oxidative stress [95].

Both pearl and finger millet hydrolyzates have been studied for their health benefits (Table 3). Tryptic hydrolyzate of pearl millet was characterized and the sequence SDRDLLGPNNQYLPK was identified, showing radical scavenging and chelating abilities [98]. A later study by the same group identified TSSSLNMAVRGGLTR and STTVGLGISMRSASVR from peptic and tryptic hydrolyzates of finger millet with similar bioactive properties [99].

#### 4.1.5. Mungbean and Sorghum Hydrolyzates

Calcium and iron-binding activities have been associated with mung bean peptides LLLGI, AIVIL, HADAD, and PAIDL, which were produced from a series of enzymatic hydrolyzates by alcalase, flavourzyme, trypsin, pancreatin, pepsin, pancreatin + alcalase, pancreatin + flavourzyme, neutral protease, or papain [100,103]. The singular use of bromelain enzyme to hydrolyze mung bean protein yielded a rich pool of hydrolyzates from which the peptides LPRL, YADLVE, LRLESF, HLNVVHEN, PGSGCAGTDL, HC, CGN, LAN, CTN, LAF, CSGD, MMGW, QFAAD, ERF, EYW, FLQL, and QFAW were identified, which showed multifunctional in vitro activity and antihypertensive action in a rat model [101,102].

White, green, or sweet sorghum hydrolyzates also have healthful potential (Table 3). Alcalase hydrolyzate of green tender sorghum was purified to synthesize VPPSKLSP, VAITLTMK, GLLGKNFTSK, LDSCKDYVME, HQTSEFK, VSKSVLVK, and TSVEIITSSK peptides with multiple activities in common biochemical assays [104], while sweet sorghum peptide TLS prepared from its alcalase protein hydrolyzate demonstrated ACE inhibitory action [106]. Papain hydrolyzate from kafirin fraction of white sorghum yielded LRQQ, QLQGV, WQPN, GLQDL, LRQQ, QLQGV, WQPN, AMCGVV, YLRQ, TPCATS, QGVAAA, AQVAQ, and QQLQ with protective effects in HepG2 cells, in addition of radical scavenging and metal chelating abilities [107]. Interestingly, by using a model of human organotypic skin cultures, fractions lower than 3 and 1 kDa were demonstrated to possess antioxidant, anti-inflammatory, and anti-aging effects against the damage induced by ultraviolet B irradiation [105].

### 4.2. Gastrointestinal Digestion

Gastrointestinal digestion induces changes to food proteins by protein breakdown and peptide release derived from the physiological systems in the gastrointestinal tract including the action of digestive enzymes [129]. Indeed, apart from the above-mentioned studies on protein enzymatic hydrolysis, the use of protocols of gastrointestinal digestion is another popular strategy explored to produce food protein hydrolyzates and bioactive peptides from underutilized grains. These various health effects are also summarized in Table 3.

Simulated gastrointestinal digestion of amaranth proteins, its sprout or germinated forms has allowed both the production of numerous protein hydrolyzates and the identification of multiple sequences including YESGSQ, GGEDE, NRPET, FLISCLL, TALEPT, HVIKPPS, SVFDEELS, DFIILE, ASANEPDEN and STHASGFFFFHPT, TIEPHGLLLPSFTSAPELIYIEQGNGITGMMIPGCPETYESGSQQFQGGEDE, GLTEVWDSNEQEF, STNYFLISCLLFVLFNGCMGEG with reported bioactivities such as anticancer and anti-inflammatory activities, ACE inhibition, radical scavenging, and DPP-IV and *α*-amylase inhibitory activities [108,109,110,111,112,113,114,115].

The use of biochemical assays has provided the examination of multiple peptides with several bioactivities derived from underutilized grains; this is an interesting screening method for food research before application of cellular systems [130]. Hence, the quinoa sequences IQAEGGLT, DKDYPK, and GEHGSDGNV showed DPP-IV, *α*-amylase, and *α*-glucosidase inhibitory activities in different enzymatic assays [116]. Simulated gastrointestinal digests of albumin and globulin fractions of chia presented ACE inhibitory, radical (ABTS, DPPH) scavenging and ferrous chelating activities [118]. Tryptic and alcalase hydrolysis of albumin and glutelin fractions beside gastrointestinal digests of buckwheat produced protein sources with inhibitory effects on the aggregation of platelets and DPP-IV [119,120]. DPP-IV, α-amylase, and α-glucosidase were inhibited by PHPATSGGGL and YVDGSGTPLT peptides obtained from bromelain hydrolyzates and simulated gastrointestinal digests of chickpea protein [121]. As for mung bean, multiple di- and tri-peptides isolated from thermolysin and gastrointestinal digests showed radical scavenging and iron-binding characteristics [124].

In cellular bioassays, antiproliferative activity has been recently discovered for amaranth proteins by independent groups [108,109,115]. Moreover, foxtail millet peptides resulting from gastrointestinal digestion, namely, SEDDPFD, REEGVLIF, EAGNKGTLSF, MGPIPSTL, EDDQMDPMAK, QNWDFCEAWEPCF, and MSHRGACGCEK, showed antioxidant and anti-inflammatory effects in Caco-2 cells [123], hence suggesting the potential of peptides released during food protein digestion to directly act as preventive compounds in the gastrointestinal tract.

In a step forward, some studies using protein digests have characterized specific peptides with antihypertensive properties in vitro which was further combined with proven health impact in animal models. This is the case of intestinal digests of quinoa, which showed antihypertensive effects in spontaneously hypertensive rats and ACE inhibitory action specifically associated with FHPFPR, NWFPLPR, and NIFRPF peptides [117]. Likewise, the specific peptides obtained from simulated digesta of cocoa seed protein, namely, LSPGGAAV, TSVSGAGGPGAGR, and TLGNPAAAGPF, showed ACE inhibitory and antihypertensive activity in a high-fat diet rat model [122].

### 4.3. Alternative Strategies

A few cases when enzymatic or gastrointestinal digestion techniques were not reported as the main proteolytic strategy, some alternative grain proteins have shown biological functions (Table 3). As an example, the buckwheat peptide (GSSEKPQQELEECQNVCRMKRWSTEMVHRCEKKCEEKFERQQR) was obtained by gene cloning and expression and found to inhibit trypsin activity and display antifungal effects against *Trichoderma koningii*, *Rhizopus* sp., and *Fusarium oxysporum* [127]. In another research effort, the purification of a 11 kDa protein from buckwheat seed extract produced the N-terminal sequence AQXGAQGGGAT which also showed antifungal activity against *B. cinerea* [128].

Alternately, amaranth protein isolates on their own could produce several metabolic effects on animal models by reducing plasma and liver cholesterol levels, increasing FRAP values and diminishing thiobarbituric acid (TBA) values in plasma and liver as well as superoxide dismutase (SOD) activity in plasma, and finally decreasing blood pressure [126]. However, this study did not identify the protein sequences causing the effects. Finally, amaranth fermentation with *Lactobacillus casei* Shirota and *Streptococcus thermophilus* 54102, in mono and combined culture, showed promising results for peptides bioactivity, as represented by ACE inhibition, radical scavenging and FRAP potential, and anti-thrombotic activities in some biochemical assays [125].

## 5. Conclusions and Future Trends

In addition of the population rise, other factors such as the expanding knowledge of the negative environmental impact of farming practices and the adverse effects associated with the excessive consumption of certain animal proteins are promoting the demand for plant-based healthy and sustainable foods. In this changing agricultural system, grains and seeds (including underutilized grains) play an important role in terms of both supporting sustainable and cost-affordable agronomic practices and maintaining healthier diets. Although the nutritional and functional properties of underutilized grains and their protein components may be already known, the evaluation of the bioactive effects of hydrolyzates and peptides thereof is now represented as an emerging area of research with promising applications facing future challenges. These innovative studies are based on their alternative value as new ingredients for the development of functional foods, infant/elderly formulas, supplemented beverages, or pharma-cosmetics.

Bioactive peptides are released from the parent protein through different approaches, being the enzymatic hydrolysis and gastrointestinal digestion the most studied. Enzymes such as pepsin, pancreatin, trypsin, papain, and alcalase, alone or in combination, have been recognized as suitable proteases to hydrolyze underutilized grains and liberate peptides with multifunctional peptides. Factors affecting the proteolysis process such as the type of enzyme and protein substrate, the substrate:enzyme ratio, and hydrolysis conditions (temperature, pH, and time), will result in different protein hydrolyzates, and consequently, different peptide profiles and a range of functional activities. Noteworthy, these bioactivities may be increased but also lowered and even lost during peptide production. Once identified, the use of in silico tools and the study of the physico-chemical properties, acid/basic/aromatic profile of amino acids, hydrophobicity, chain length, and molecular weight of bioactive peptides, along with the application of docking methods and omics analysis will help at deciphering of the mechanisms of action involved in their attributed physiological effects and health impacts. Also, stability, absorption and bioavailability models must be considered to provide additional information needed to confirm the potential of these bioactive peptides as novel functional food and nutraceutical ingredients.

## Figures and Tables

**Table 1:** Nutritional, biological and techno-functional impact of the incorporation of underutilized grains in functional foods.

Grain Species	Type of Food	Nutritional Composition	Biological Activity	Physicochemical and Techno-Functional Properties	Reference
Amaranth	Fortified pasta	Enhancement of fiber and protein contents Increase of β-carotene, iron, and zinc contents compared with white cassava-amaranth pasta	Improvement of the antioxidant (DPPH) capacity	Reduction of cooking time and gruel solid loss in amaranth-fortified pasta	[10]
	Enriched cookies	---	ACE inhibition by serum from mice fed enriched cookies Reduction of blood pressure in hypertensive rats	---	[11]
	Functional cookies	Increase of total protein, ash and flavonoids contents compared with control cookies	Increase of antioxidant (ABTS) capacity compared with control cookies	---	[12]
	Enriched pasta	---	Antihypertensive properties in hypertensive rats	Decrease of optimum cooking time and cooking loss	[13]
	Puffed snacks	High protein, iron, zinc, and dietary fiber content	---	---	[14]
*A. caudatus*	Muffin	Increase of nutritional (fiber, fat, protein) quality	Increase of total phenolics and flavonoid content, and antioxidant (ABTS and DPPH) activities	Improvements of color, texture characteristics	[15]
	Multigrain bread	Increase of ash, fiber, and protein contents and reduction of carbohydrate content	Exhibition of significant hydroxyl radical scavenging ability, Fe2+ chelation and inhibition of Fe2+-induced lipid peroxidation Increase of α-amylase and α-glucosidase inhibitory abilities, and decrease of glycemic indices compared to white flour bread (control)	---	[16]
*A. hypochondriacus*	Elbow-type pasta	Increase of protein, crude fiber and ash content in flour and dry leaves pastas Increase of iron, zinc, magnesium, and potassium in dry leaves pasta	Loss of antioxidant (FRAP and ORAC) capacity after cooking (low decrease in dry leaves pasta)	Decrease of cooking time, increase of cooking loss percentage, and decrease of luminosity values compared with semolina control pasta	[17]
	Lemon sorbet	---	Release of antithrombotic peptides during simulated gastrointestinal digestion	Increase of foaming properties	[18]
	Amaranth-based beverage	Good nutritional quality (high value proteins, lipids, and carbohydrates, and high content of soluble fiber)	---	---	[19]
*A. caudatus*, quinoa (*C. quinoa* Willd.), and black chia (*S. hispanica* L.)	Bread	Significant increase in protein amount, ash, lipids, and crumb firmness compared to wheat bread Similar calorie value between control and fortified formula	---	Decrease of loaf-specific volume in comparison to control bread	[20]
*A. viridis*, sorghum (*S. bicolor*), and wholesome sesame (*S. indicum*)	Spaghetti pasta	Significant increase of protein, ash, fat, fiber, calcium, magnesium, and zinc contents compared to control (100% wheat flour pasta)	Increase of alkaloids, total phenolic, flavonoids, DPPH, and FRAP relative to control (100% wheat flour pasta)	---	[21]
Buckwheat	Bread	Increase of iron content	---	Decrease of the porosity and specific loaf volume of the bread	[22]
	Biscuits	Identification of total phenolic compounds (p-coumaric, sinapic, syringic, vanillic, protocatechuic acids, kaempherol, quercetin, apigenin, and orientin)	Decrease of ACE inhibitory activity that was reverted by simulated gastrointestinal digestion Partial correlation of ACE inhibitory activity with the content of total phenolic compounds	---	[23]
	Biscuits	---	Increase of bio-accessible anti- antioxidant/reducing capacity, AGEs activity, and total phenolics content after simulated digestion of biscuits Contribution of the bio-accessible phenolic antioxidants to the AGEs formation-inhibitory activity of biscuits	---	[24]
Buckwheat (*F. esculentum* Moench)	Beef burger	Increase of magnesium, phosphorus, iron, and zinc contents in comparison with control burgers	---	Reduction of oil absorption and water holding capacity in comparison with soy protein and bread crumb (control) burgers Increase of shelf-life stability	[25]
	Noodle	Low concentration of total phenolics and flavonoids	Low antioxidant (FRAP, ABTS, and DPPH) capacity	High tensility and low adhesiveness	[26]
Buckwheat (*F. sagittatum* Gilib)	Functional desserts	Increase of dietary fibers content	Increase of antioxidant capacity	---	[27]
Tartary buckwheat (*F. tartaricum*)	Noodle	High concentration of total phenolics and flavonoids	Increase of (FRAP, ABTS, and DPPH) antioxidant capacity	Low tensility and increase of adhesiveness	[26]
Tartary buckwheat (*F. tataricum* Gaertn.) and chia (*S.* *hipanica* L.)	Bread	Increase of protein, insoluble dietary fibers, ash, and alpha-linolenic acid Reduction of energy and carbohydrate contents compared to the control (wheat) bread	Improvement of the total antioxidant (FRAP, ORAC) capacity of the buckwheat bread	---	[28]
Chia (*S. hipanica* L.)	Cookies	Increase of protein and dietary fiber content	---	---	[29]
	Beef burger	Increase of PUFAs and polyphenols contents	Increase of (ORAC, ABTS, DPPH) antioxidant capacities, and reduction of MDA values Increase of polyphenol bio-accessibility, antioxidant capacity, and MDA after simulated gastrointestinal digestion	---	[30]
	Bread	Increase of PUFAs (mainly linolenic acid) content and reduction of SFA and MUFA content Reduction of n-6/n-3 ratio in special breads prepared with kinako flour and chia	---	---	[31]
	Functional yogurt	Increase of crude protein, lipids, dietary fiber, PUFAs (mainly n-3), and mineral content	---	During storage, the yogurt had adequate amounts of lactic acid bacteria, and Bifidobacteria (probiotic characteristics)	[32]
	Functional yogurt	---	Increase of radical (DPPH and ABTS) scavenging activity Inhibition of LPS-induced production of H_2_O_2_ in human colon cells	Acceleration of the fermentation rate and growth of lactic acid bacteria Improvement of syneresis, and water-holding capacity	[33]
	Sweet cookies	---	Increase of the polyphenol content and the antioxidant (FRAP and ABTS) capacity Few polyphenols released from the food matrix during gastrointestinal digestion, and absorbed by passive diffusion in the small intestine Greater release of polyphenols and increase of antioxidant capacity during colonic fermentation Prebiotic effects of chia polyphenols	---	[34]
	Bread	Increase of protein, lipids, and minerals content Increase of linoleic acid	Reduction of glycemic index compared to control (wheat flour) bread	---	[20]
	Pineapple jam	Increase of protein and crud fiber in comparison with control jam	---	Significant differences in texture, but not in spreading properties, compared with control jam	[35]
	Cake	Increase of protein, lipid, and ash content in comparison with control cake	---	Decrease of the specific volume and color parameters of the cakes	[36]
	Bread	Reduction of water activity, and equal amount of moisture compared with the control	---	Improvement of gas retention in dough and cut the time required to reach maximum dough development Delay in hardness and water loss during storage of breads	[37]
	Kulfi dessert	Increase of protein and fiber content	---	---	[38]
	Corn tortillas	Increase of protein, lipids, and total dietary fiber content compared with control tortilla	Reduction of enzymatic starch hydrolysis rate and predicted glycemic index	---	[39]
	Gluten-free noodle	Increase of protein and fat, phytic acid and phytate phosphorus contents Increase in the amounts of Ca, P, K, Mg, Fe and Zn	Increase in antioxidant activity (DPPH) and total phenolic content	Significant rise in volume increase and weight increase values	[40]
Lupin (*L. albus* L.)	Biscuits	Increase of protein, lipid, fiber and ash content, and reduction of carbohydrate content compared with control (wheat flour) biscuits Rise of all amino acids content	---	Improvement of quality scores	[41]
	Sweet cookies	Low values of water activity and moisture content	---	Higher firmness but reduced impact on the shape parameters, namely in area and thickness Decrease of the lightness	[42]
Lupin (*L. angustifolius*)	Beef sausage	Increase of dietary fiber and reduction of fat content	---	Increase of the meat emulsion stability and decrease of cooking loss Greater adhesiveness	[43]
Quinoa (*C. quinoa* Willd.)	Beef burger	Increase of Mg, P, Fe, and Zn contents in comparison with control burgers	---	Reduction of oil absorption and water holding capacity in comparison with soy protein and bread crumb (control) burgers Increase of shelf-life stability	[25]
	Bread	Increase in protein, dietary fiber, thiamine, Mg, Fe, and P	---	---	[44]
	Bread	Increase of protein, fiber, vitamins, mineral, and essential amino acids content, and reduction of starch content	---	Decrease of the lightness and redness, and increase of yellowness	[45]

ABTS: 2,2′-azinobis(3-ethylbenzothiazoline-6-sulphonic acid) diammonium salt; ACE: angiotensin converting enzyme; AGEs: Advanced glycation end products; DPPH: 2,2-diphenyl-1-picryl-hydrazyl; FRAP: ferric reducing antioxidant power; MDA: malondialdehyde; MUFAs: monounsaturated fatty acids; ORAC: oxygen radical antioxidant capacity; PUFAs: polyunsaturated fatty acids; SFA: saturated fatty acids.

**Table 2:** Sensorial analysis of functional foods formulated with addition of underutilized grains.

Food Group	Grain Specie	Type of Addition	Sensorial Analysis	Reference
Bread	Amaranth (*A. caudatus*), quinoa (*C. quinoa* Willd.), and black chia (*S. hispanica* L.)	Flours	Higher nutritional and functional indexes and similar overall acceptability in fortified bread	[20]
	Buckwheat	Buckwheat flour	Good scores for quality indicators of bread (taste, color, aroma, texture, and bread surface)	[22]
	Quinoa (*C. quinoa* Willd.)	Quinoa flour	Higher sensory acceptability of quinoa-based pan bread in comparison to the control	[45]
Pasta	Amaranth	Amaranth protein hydrolyzate by alcalase	Negative impact on overall acceptability and taste of enriched pasta	[13]
	Amaranth (*A. hypochondriacus*)	Amaranth flour/dry leaf/semolina	Decrease of luminosity values of elbow-type pasta compared with semolina control pasta	[17]
	Amaranth (*A. viridis*), sorghum (*S. bicolor*), and wholesome sesame (*S. indicum*)	Flours	Superiority in aroma, taste, and acceptability of spaghetti product indexes relative to the control	[21]
	Common buckwheat (*F. esculentum* Moench)	Buckwheat flour	Good sensory attributes of noodles	[26]
	Tartary buckwheat (*F. tartaricum*)	Buckwheat flour	Bitter taste of noodles	[26]
	Chia (*S. hipanica* L.)	Chia flour	Improvement of taste and odor scores of gluten-free noodles	[40]
			No differences in sensory attributes compared with control corn tortilla	[39]
Confection	Amaranth	Amaranth protein hydrolyzate by alcalase	Intensification of yellow-brown color in enriched cookies	[11]
		Amaranth flour + roba1 beans + maize grain + fresh, orange-fleshed sweet potato	Moderate sensory acceptance of puffed snacks by consumers	[14]
	Amaranth (*A. caudatus*)	Amaranth flour + black rice flour	Improvement of sensory attributes of supplemented muffin	[15]
	Buckwheat (*F. sagittatum* Gilib)	Buckwheat hull and derived melanin	Good sensory acceptance of functional desserts	[27]
	Chia (*S. hipanica* L.)	Chia seeds and flour	Greater acceptance for cookies descriptors of good texture, good color, good odor, sweet taste, good taste, and pleasant taste	[29]
		Defatted chia flour	Higher sensory acceptance in comparison with control cookies	[34]
		Chia flour and hydrogenated vegetable fat	Decrease of color parameters in comparison with control cake	[36]
		Chia flour	Good sensory acceptance of kulfi dessert	[38]
		Chia seed	Differences in general acceptability and sensory evaluation (flavor, color, and texture) compared with control jam, with negative impact at higher levels of addition	[35]
	Lupin (*L. albus* L.)	Lupin flour	Improvement of score of color, crust appearance, texture, aroma, taste, and overall acceptability of biscuits	[41]
		Lupin flour + oat + buckwheat	Higher firmness and improved sensory attributes of sweet cookies	[42]
Beverages and yogurt	Amaranth (*A. hypochondriacus*)	Amaranth protein isolate	Good acceptability assay in terms of airy, creamy, and healthy attributes of lemon sorbet	[18]
	Chia (*S. hipanica* L.)	Chia seeds + strawberries	Sensory acceptance inversely proportional to the addition of chia but increased with the addition of strawberry	[32]
Meat	Buckwheat (*F. esculentum* Moench)	Buckwheat flour	Increase of overall acceptability, taste attributes, and shelf-life stability of beef burger	[25]
	Chia (*S. hipanica* L.)	Chia seeds	Good sensorial acceptance of beef burger in hedonistic tests	[30]
	Lupin (*L. angustifolius*)	Lupin flour	No significant difference in appearance, aroma, flavor and overall acceptability between control and lupin-enriched beef sausage	[43]
	Quinoa (*C. quinoa* Willd.)	Quinoa flour	Increase of overall acceptability, taste attributes, and shelf-life stability of beef burger	[25]

## Data Availability

Not applicable.
